# Nutrition guidance within a multimodal intervention improves diet quality in prodromal Alzheimer’s disease: Multimodal Preventive Trial for Alzheimer’s Disease (MIND-AD_mini_)

**DOI:** 10.1186/s13195-024-01522-8

**Published:** 2024-07-03

**Authors:** Nicholas Levak, Jenni Lehtisalo, Charlotta Thunborg, Eric Westman, Pia Andersen, Sandrine Andrieu, Laus M. Broersen, Nicola Coley, Tobias Hartmann, Gerd Faxén Irving, Francesca Mangialasche, Tiia Ngandu, Johannes Pantel, Anna Rosenberg, Shireen Sindi, Hilkka Soininen, Alina Solomon, Rui Wang, Miia Kivipelto

**Affiliations:** 1 Division of Clinical Geriatrics, Department of Neurobiology, Care Sciences and Society, Karolinska Institute, Center for Alzheimer Research QA32, Karolinska Vägen 37 A, 171 64 Solna, Sweden; 2 Theme Inflammation and Aging, Karolinska University Hospital, Stockholm, Karolinska Vägen 37 A, 171 64 Solna, Sweden; 3 Population Health Unit, Department of Public Health and Welfare, Finnish Institute for Health and Welfare, Helsinki, Finland; 4 Department of Neurology, Institute of Clinical Medicine, University of Eastern Finland, Yliopistonranta 8, 70210 Kuopio, Finland; 5 Department of Caring Sciences, Faculty of Health and Occupational Studies, University of Gävle, Gävle, Sweden; 6 Department of Neuroimaging, Centre for Neuroimaging Sciences, Institute of Psychiatry, Psychology and Neuroscience, King’s College London, London, England; 7 Department of Clinical Epidemiology and Public Health, UMR 1295, CHU de Toulouse, and Aging Research Team, CERPOP Inserm, Jules Guesde, 31000 Toulouse, France; 8 IHU HealthAge, Toulouse, 31059 France; 9 Danone Research & Innovation, Utrecht, Netherlands; 10 German Institute for Dementia Prevention (DIDP), Saarland University, 66424 Homburg, Germany; 11 Department of Experimental Neurology, Medical Faculty, Saarland University, 66424 Homburg, Germany; 12 Institute of General Practice, Goethe University Frankfurt a.M., Frankfurt, Germany; 13 The Ageing Epidemiology (AGE) Research Unit, School of Public Health, Imperial College London, St Mary’s Hospital, Norfolk Place, London, W2 1PG UK; 14 Department of Physical Activity and Health, the Swedish School of Sport and Health Sciences, 114 86 Stockholm, Sweden; 15 Wisconsin Alzheimer’s Disease Research Center, School of Medicine and Public Health, University of Wisconsin, 600 Highland Ave J5/1 Mezzanine, Madison, WI 53792 USA; 16 Institute of Public Health and Clinical Nutrition, University of Eastern Finland, Yliopistonranta 8, 70210 Kuopio, Finland

**Keywords:** Nutrition, Alzheimer’s disease, Dementia, Multimodal trial, Lifestyle, Prodromal Alzheimer’s disease

## Abstract

**Background:**

Multimodal lifestyle interventions can benefit overall health, including cognition, in populations at-risk for dementia. However, little is known about the effect of lifestyle interventions in patients with prodromal Alzheimer’s disease (AD). Even less is known about dietary intake and adherence to dietary recommendations within this population making it difficult to design tailored interventions for them.

**Method:**

A 6-month MIND-AD_mini_ pilot randomized controlled trial (RCT) was conducted among 93 participants with prodromal AD in Sweden, Finland, Germany, and France. Three arms were included in the RCT: 1) multimodal lifestyle intervention (nutritional guidance, exercise, cognitive training, vascular/metabolic risk management, and social stimulation); 2) multimodal lifestyle intervention + medical food product; and 3) regular health advice (control group). Adherence to dietary advice was assessed with a brief food intake questionnaire by using the Healthy Diet Index (HDI) and Mediterranean Diet Adherence Screener (MEDAS). The intake of macro- and micronutrients were analyzed on a subsample using 3-day food records.

**Results:**

The dietary quality in the intervention groups, pooled together, improved compared to that of the control group at the end of the study, as measured with by HDI (*p* = 0.026) and MEDAS (*p* = 0.008). The lifestyle-only group improved significantly more in MEDAS (*p* = 0.046) and almost significantly in HDI (*p* = 0.052) compared to the control group, while the lifestyle + medical food group improved in both HDI (*p* = 0.042) and MEDAS (*p* = 0.007) during the study. There were no changes in macro- or micronutrient intake for the intervention groups at follow-up; however, the intakes in the control group declined in several vitamins and minerals when adjusted for energy intake.

**Conclusion:**

These results suggest that dietary intervention as part of multimodal lifestyle interventions is feasible and results in improved dietary quality in a population with prodromal AD. Nutrient intakes remained unchanged in the intervention groups while the control group showed a decreasing nutrient density.

**Trial registration:**

ClinicalTrials.gov NCT03249688, 2017–07-08.

**Supplementary Information:**

The online version contains supplementary material available at 10.1186/s13195-024-01522-8.

## Introduction

Alzheimer’s disease (AD) and dementia are important and multifaceted public health challenges in an aging population. Given that the number of dementia cases is projected to triple by 2050, there is a growing need for effective measures to prevent and slow the disease process [1, 2]. Although there have been encouraging advancements in the use of new anti-amyloid drugs [3–5], these disease modifying drugs for AD are not suitable for everyone. Observational studies support multifactorial pathological processes in dementia, with a healthy lifestyle linked to reduced cognitive decline. The Lancet Commission identified 12 modifiable risk factors that could account for approximately 40% of all dementia cases [6]. These risk factors for cognitive decline are interconnected and can have synergistic effects, and they are linked to many factors that were not listed like diet. Although a healthy diet is regarded as a crucial component of healthy aging, the relationship between diet and cognitive function is still evolving, with often inconsistent or conflicting findings [7]. Diet should be considered a complex, multifaceted aspect that includes both beneficial and detrimental components, which can influence the brain either directly or indirectly [8].

The prodromal stages of AD that occur before clinically significant cognitive impairment present valuable opportunities to prevent or delay dementia onset [9]. Currently there is limited knowledge about lifestyle and especially diet in this group. Already in the early stages of disease, patients are at increased risk of being or becoming malnourished [10–14]. Moreover, irrespective of their overall nutritional status, patients with early AD (i.e. mild cognitive impairment (MCI) due to AD) [15] have an elevated need for energy containing nutrients [16]. AD is often associated with unintentional weight loss, which results in declining functional capacity [17].

Currently it is unclear which dietary factors are the most effective in preventing or delaying dementia, and whether there are differences in nutritional needs between the disease stages. Observational studies suggest a protective association between specific nutrients (folate, vitamin D and certain lipids) or food groups (vegetables, fruits, nuts, legumes, berries, fish, and seafood) and cognition in older people [18]. Additionally, dietary patterns, particularly the Mediterranean, DASH (Dietary Approach to Stop Hypertension), and MIND (Mediterranean-DASH Intervention for Neurodegenerative Delays) have been related to improved cognitive function and a reduced risk of developing AD [19]. Observational studies are prone to reverse causation, confounding, and overadjustment [19]. However, only one larger-scale intervention trial has been completed for the Mediterranean diet [20], with positive results; and one for the DASH diet [21], and one for the MIND diet with no effect on cognition [22]. Diet intervention trials designed primarily to prevent cognitive decline, especially for people with prodromal AD, are limited and previous trial evidence is inconsistent [22–24]. However, the LipiDiDiet trial has shown promising results using the medical food product Fortasyn Connect (Souvenaid™) in the targeted prodromal AD population [25, 26]. Nutritional interventions have been highlighted to be particularly important in the prodromal stages due to decreasing blood circulatory nutrient status with subsequent nutritional insufficiencies [27]. Hence, there is a need for intervention studies to identify the ideal dietary composition, evaluate feasibility, and assess adherence among individuals with prodromal AD.

The FINGER study, a randomized controlled trial targeting at-risk older individuals demonstrated the potential benefits of a multimodal lifestyle intervention in preventing cognitive decline [28]. The dietary component of the FINGER intervention was proven to be successful in promoting healthy dietary changes related to improvements in cognition [29, 30]. Adopting the results from the FINGER study, the Multimodal Preventive Trial for Alzheimer’s Disease (MIND-AD_mini_) trial explored the feasibility of the FINGER intervention in individuals with prodromal AD by integrating lifestyle components with the medical food product Fortasyn Connect [31]. The aim of this study was twofold. First, we aim to evaluate overall diet quality and intervention-related changes in diet quality using dietary indices within a multimodal lifestyle intervention for patients with prodromal AD. In addition to the pre-specified secondary outcome, the MEDAS score [32] reflecting adherence to the Mediterranean diet, we utilized the Healthy Diet Index [33], a tool formulated and validated to measure adherence to a health-promoting diet particularly in Nordic countries. Second, we assessed the level of nutrient intake and changes within the Swedish and Finnish study population.

## Methods

### Recruitment of participants

Ninety-three participants with prodromal AD, according to the International Working Group-1 criteria [34], were recruited from memory clinics in Stockholm, Sweden, and Toulouse, France, through media advertisements in Frankfurt, Germany, and from previous research cohorts in Kuopio, Finland, via the university hospital neurology clinic. Participants were older adults between 60 and 85 years of age with an MMSE score of ≥ 24 and an eligible study partner, who could help facilitate the intervention components, as well as room for lifestyle improvement according to the MIND-AD_mini_ study specific healthy lifestyle index. Further inclusion criteria have been presented in the study protocol [31].

Exclusion criteria included preexisting dementia diagnosis or major depressive disorder according to the Diagnostic and Statistical Manual of Mental Disorders, Fourth Edition (DSM-IV); concomitant severe disease; intake of supplements for vitamin B6, vitamin B12, folic acid, vitamin C, or vitamin E exceeding 200% of the recommended daily intake (RDI)(above recommendations in NNR [35]) unless prescribed by a physician; or use of omega-3 preparations (> 500 mg eicosapentaenoic acid (EPA) + docosahexaenoic acid (DHA) per day).

### Trial design

A complete description of the study can be found in the study protocol [31]. The study was a randomized, controlled, parallel-group feasibility study with three groups: a lifestyle intervention only group, a lifestyle + medical food group, and a control group receiving standard health advice. The CONSORT checklist and flowchart, feasibility and adherence to the intervention components as well as dropouts have been reported earlier [36]. In brief, the intervention consisted of an adapted FINGER model with five modalities: healthy dietary advice, physical exercise, cognitive training, social activity, and monitoring and treatment of cardiovascular risk factors. The intervention lasted for 6-months. The medical food product was a 125 ml once-a-day milk-based drink with the multinutrient combination Fortasyn Connect. The combination contains elevated levels of the long-chain omega-3 fatty acids EPA and DHA, uridine monophosphate, choline, vitamins B12, B6, C, and E, folic acid, phospholipids, and selenium. Participants receiving the medical food product were instructed to fill in a written record on their adherence and to bring in empty bottles for counting. The study was approved by the ethics review boards in each country and all participants provided written informed consent.

### Assessment

Study visits have previously been described [31]. In short, participants completed a demographic questionnaire, and underwent physical and cognitive measurements. A trained study nurse measured height, weight, hip (at the level of the largest lateral extension of the hips), and waist (midpoint between lowest rib and the iliac crest) circumference whilst participants were standing [37]. Blood pressure was measured twice and the mean of the two measurements was used. The main dietary data source was a brief food intake questionnaire (FIQ), which consisted of 48 multiple-choice questions (frequency and quality), to evaluate overall diet quality in all countries. The FIQ combines questions from two previously validated questionnaires [38, 39]. Additionally, data were also obtained from a 3-day food record of three days, i.e., two weekdays and one weekend day (Friday-Sunday), to calculate nutrient intakes. Only food records from Sweden (*n* = 21) and Finland (*n* = 29) were available and used to calculate nutrient intake. Participants received written instructions to document all the foods and beverages they consumed, specifying the type, brand, and method of preparation, and utilizing household measures. They were also asked to record any vitamin and mineral supplements they might have taken with dosage, interval, and brand name.

### Dietary intervention

Participants in the intervention groups received dietary counseling through three group sessions and three individual sessions with the study dietitian. Individual sessions were at baseline, 3 months, and 4 months and were scheduled to be 30 min long. Dietary guidance were tailored according to and improved on the participant’s previous diet. The group sessions were intended to provide more information, motivation, and resources to support participants in making and maintaining dietary changes. Sessions shared themes between sites to ensure similar content and intensity while allowing national adaptations. Themes included e.g. dietary fat quality; vitamins, minerals, and antioxidants; grocery shopping and nutrition labels; and alcohol consumption. The study partner was encouraged to attend group and individual sessions.

Recommendations were based on the latest national recommendations at the time, e.g. the Nordic Nutrition Recommendations 2012 [35] in Finland and Sweden. Participants in the intervention groups were encouraged to consume ≥ 500 g of vegetables and fruit/day, ≥ 2 portions of fish or shellfish each week, rapeseed or olive oil when cooking instead of butter, wholegrain cereal products instead of refined ones, and limited sugar and sweet intake. The nutrient intake goals included 15–20 E% protein; 25–40 E% fat where of saturated fatty acids (SFA) < 10 E%; monounsaturated fatty acids (MUFA) 10–20 E%; polyunsaturated fatty acids (PUFA) 5-10 E% of which n-3 > 1 E%; trans-fatty acids as low as possible; 45–60 E% carbohydrates of which refined sugar < 10 E%; 25–35 g dietary fiber/day; salt (NaCl) ≤ 6 g/day; and alcohol < 5 E%. All participants in the intervention groups were advised to take an additional vitamin D supplement of 10–20 µg/day.

In addition to diet intervention, both active intervention groups were invited twice weekly for physical exercise in the group and cognitive training. They also met the study physician for management of their risk factors.

### Definition of healthy diet

Changes in dietary quality within and between groups were analyzed using the modified version of the Healthy Diet Index (HDI) [33] and the modified Mediterranean Diet Adherence Screener (MEDAS) [32] score. Both are calculated from the brief FIQ. The MEDAS score was a prespecified secondary outcome in the MIND-AD trial and HDI was introduced as an exploratory outcome more suited for Nordic countries. FIQ, being developed and validated in Finland [33]. The HDI consists of seven weighted domains: meal patterns 0–10 points, grains 0–20 points, fruits and vegetables 0–20 points, fats 0–15 points, fish and meat 0–10 points, dairy 0–10 points, and snacks and treats 0–15 points; for a total of 100 points. However, data on the use of cream for cooking and consumption of frankfurters were not available; thus the maximum achievable HDI score was 97 points. The MEDAS is a validated tool that consists of 14 questions, each with 1 point. It was developed as a short screening tool to assess adherence to the Mediterranean diet. Components related to fat quality were modified so that also other vegetable oils were accepted as positive components, e.g. rapeseed oil which is typically used instead of olive oil in the Nordic countries. Margarine was also excluded for the question regarding bread spreads due to it being recommended over butter because its favorable fatty acid composition. Dietary record data were recorded by dietitians and analyzed using a software program (DietistNet Pro) with each corresponding country’s national food composition database (Livsmedelsverkets database 2021–05-03 for Sweden and Fineli database release 20, 2019–07-27 for Finland). Nutritional intake was analyzed as the mean intake of the 3 days including any dietary supplements. Nutrient intake from medical food products is analyzed together with food records but consumption is not recorded in the FIQ or considered in the indices. Participants in the intervention group completed their baseline food records after medical product use was initiated, and thus, the baseline records included intake from the product.

### Statistical analysis

Baseline characteristics were reported using descriptive statistics and compared between intervention arms (control, lifestyle, lifestyle + medical food) using ANOVA for continuous variables and chi-square tests for categorical variables. We carried out a mixed linear effect model to investigate the effect of the intervention on MEDAS, HDI, and nutrient intake, by introducing the interaction term between the intervention groups and time. We further examined the changes in MEDAS, HDI, and nutrient intake, within each intervention group by examining whether the slope estimated for the linear mixed effect was significantly different from 0. This is to capture the trajectories of nutrient intake, HDI, and MEDAS within each intervention group. The models were adjusted for age, sex, and education. The results are reported as beta-coefficients and standard errors (SEs). Nutrient intake was examined in two ways: as a crude amount and nutrient density. For energy-containing nutrients we calculated proportion of energy intake for each type of macronutrient, and for micronutrients we used unit per 1 megajoule of energy. To ensure accurate analysis, the data was log-transformed to reduce skewness, but for ease of understanding, the results are presented in their original form, along with the statistical significance (*P* values) derived from the transformed data. Since the MIND-AD_mini_ trial aimed to measure the feasibility and adherence of the multimodal intervention in individuals with prodromal AD, formal sample size calculations were not conducted. *p* < 0.05 was considered to indicate statistical significance.

Data analysis was performed using R statistical software version 4.1.3 (R Core Team, 2023) with the tidyverse [40] package used for data management and visualization, the lme4 [41] and lmerTest [42] packages for linear mixed modeling.

## Results

### Participant characteristics

Table 1 Baseline characteristics and anthropometric measurements of all randomized participants.

**Table 1:** Baseline characteristics and anthropometric measurements of all randomized participants

Characteristic	Control, (*n* = 30)	Lifestyle intervention, (*n* = 32)	Lifestyle intervention + medical food, (*n* = 31)	*P*
Age, years, mean (SD)	73.7 (5.7)	72.4 (6.3)	72.7 (6.8)	0.698
Sex female, n (%)	14 (47.7)	21 (65.6)	15 (48.4)	0.249
MMSE, median (IQR)	28 (27–29)	28 (26–29)	28 (27–29)	0.674
Education, years, mean (SD)	13.7 (3.2)	12.3 (3.7)	12.5 (4.0)	0.303
Smoking, %				
Never	23 (76.7)	27 (84.3)	24 (77.4)	
Former	4 (13.3)	4 (12.5)	6 (19.3)	
Current	3 (10.0)	1 (3.1)	1 (3.2)	
Dropouts, n %	1 (3,3)	4 (12,5)	3 (9,7)	
BMI, kg/m^2^, mean (SD)	26.1 (3.8)	25.7 (3.8)	26.6 (4.1)	0.661
Waist, cm, mean (SD)	95.4 (8.8)	93.0 (11.8)	96.4 (14.7)	0.527
Waist/hip-ratio, mean (SD)	0.94 (0.08)	0.91 (0.09)	0.93 (0.10)	0.611
SBP, mmHg, mean (SD)	143 (20)	140 (15)	145 (17)	0.498

All *P*-values > 0.2

*Abbreviations*: *MMSE* Mini Mental State Examination, *IQR* Interquartile range, *BMI* Body Mass Index, *SBP* Systolic Blood Pressure

The participants were on average 72.9 years old and 52.3% were female with no differences between the intervention groups and the control group at baseline, as shown in Table 1. No changes in these characteristics, except for systolic blood pressure, were found between baseline and the 6-month follow-up. We observed a decrease in systolic blood pressure in the lifestyle intervention + medical food group (β = -10.438, SE = 3.295, p for within group change < 0.001), but not in the other groups. Overall, 79 (84.9%) participants in all countries had FIQ from both visits, and 50 (75.7%) participants in Sweden and Finland had both 3-day food records FIQ.

### Healthy diet index

**Fig. 1:**
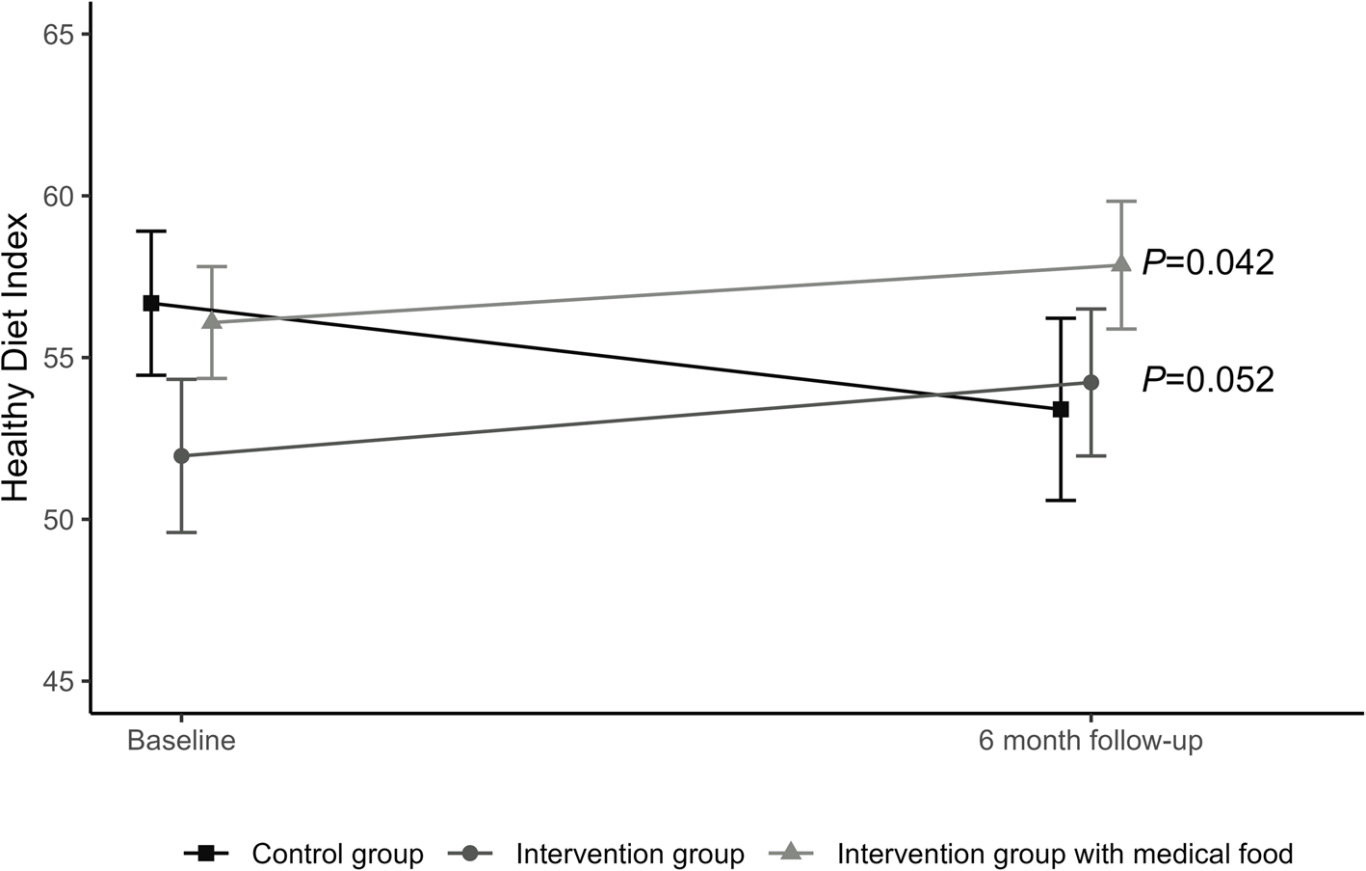
Change in Healthy Diet Index over intervention period

There was a moderate positive correlation between the HDI and MEDAS score, with a Pearson correlation coefficient of r(59) = 0.552 and *p* < 0.001 at baseline. As detailed in Tables 2 and 3 both the lifestyle intervention group and the lifestyle intervention + medical food group exhibited favorable changes in both the HDI and MEDAS scores compared to those of the control group at the 6-month follow-up. Figure 1 shows the change in the HDI over the intervention. For the HDI, the differences in changes between the lifestyle intervention only group and the control group were marginal significant (β = 4.890, SE = 2.462, *p* = 0.052) and were significant for the lifestyle + medical food group compared to the control group (β = 5.344, SE = 2.568, *p* = 0.040. When the lifestyle intervention groups were pooled together, their changes at the 6-month follow-up were greater than those in the control group for both the HDI (β = 4.987, SE = 2.191, *p* = 0.026) and MEDAS (β = 1.313, SE = 0.484, *p* = 0.007) groups.

**Table 2:** Overview of HDI and MEDAS score for all study groups and the pooled intervention group

		Control (*n* = 28)	Lifestyle Intervention (*n* = 28)	P*	Lifestyle + Medical Food (*n* = 29)	P*	Pooled Intervention Groups (*n* = 57)	P*
HDI								
Median (IQR)	Baseline	59 (52–65)	50 (42–58)		58.0 (51–64)		56.0 (48–63)	
Mean Δ (SE)	6-m	-2.4 (1.6)	1.9 (1.5)	0.052	3.1 (1.2)*	0.040	2.5 (1.0)*	0.027
MEDAS								
Median (IQR)	Baseline	6.0 (4–7)	6.0 (5–8)		5.5 (5–7)		6.0 (5–8)	
Mean Δ (SE)	Δ 6-m	-0.4 (0.3)	0.5 (0.4)*	0.045	1.0 (0.4)*	0.007	0.7 (0.3)*	0.007

Mixed linear regression for between group differences and mean change from baseline. Lower/upper limit: HDI 0–97, MEDAS score 0–14

^*^ *P* < 0.05 for change between intervention groups and control group

**Table 3:** Intake and changes in intake of energy, macro- and micronutrients for the Swedish and Finnish study population (Mean values with their standard errors)

		Control (*n* = 18)	Lifestyle intervention (*n* = 14)		Lifestyle + medical food (*n* = 16)		NNR recommended intake
		Mean	Mean	*P*	Mean	*P*	
Energy, MJ	Base	8.4 (2.8)	7.7 (2.)		8.5 (1.6)		
	Δ 6-m	-0.3 (1.8)	0.0 (1.9)	0.520	-0.2 (1.7)	0.900	
Totalt fat, E%	Base	36 (6)	36 (5)		34 (7)		25–40
	Δ 6-m	2 (7)	3 (6)	0.556	2 (7)	0.790	
SFA, E%	Base	14 (4)	13 (3)		12 (4)		< 10
	Δ 6-m	1 (5)	1 (3)	0.765	0 (4)	0.545	
MUFA, E%	Base	13 (2)	14 (3)		12 (3)		10–20
	Δ 6-m	0 (3)	0 (4)	0.873	1 (3)	0.864	
PUFA, E%	Base	6 (2)	6 (2)		7 (2)		5–10
	Δ 6-m	0 (3)	8 (4)	0.170	8 (3)	0.077	
n-3 PUFA, E%	Base	1.5 (0.7)	1.2 (0.4)		1.8 (0.6)		≥ 1
	Δ 6-m	-0.1 (0.7)	0.3 (0.8)	0.122	0.6 (1.0)	0.002*	
n-6 PUFA, E%	Base	4.1 (1.5)	3.7 (0.9)		4.0 (2.0)		
	Δ 6-m	0.3 (2.3)	1.2 (3.5)	0.324	0.7 (2.3)	0.268	
Carbohydrate, E%	Base	40 (6)	42 (7)		45 (7)		45–60
	Δ 6-m	-1 (7)	-1 (7)	0.798	-2 (7)	0.336	
Sucrose, E%	Base	7 (3)	7 (4)		7 (3)		< 10
	Δ 6-m	0 (3)	1 (7)	0.480	7 (4)	0.911	
Fiber, g †	Base	26.0 (9.1)	20.1 (5.6)		27.2 (7.1)		F ≥ 25, M ≥ 35
	Δ 6-m	-3.8 (6.8)	2.3 (7.4)	0.036*	-0.5 (7.3)	0.209	
Protein, E%	Base	17.5 (3.7)	17.4 (3.2)		17.4 (2.3)		15–20
	Δ 6-m	-0.8 (3.6)	-0.4 (2.9)	0.508	-0.1 (3.1)	0.983	
Alcohol, E% †	Base	4 (4)	2 (2)		1 (2)		< 5
	Δ 6-m	-1 (3)	-1 (1)	0.874	-0 (2)	0.499	
Calcium, mg	Base	1053 (372)	824 (357)		1088 (420)		800
	Δ 6-m	-53 (244)	50 (412)	0.392	-36 (377)	0.671	
Potassium, mg	Base	3567 (1034)	3311 (996)		3767 (642)		F = 3.1, M = 3.5
	Δ 6-m	-432 (824)	-105 (861)	0.225	-56 (1162)	0.452	
Phosphorus, mg	Base	1618 (520)	1354 (400)		1642 (324)		600
	Δ 6-m	-172 (324)	46 (410)	0.115	13 (392)	0.318	
Vitamin C, mg	Base	133 (87)	136 (83)		150 (57)		75
	Δ 6-m	-33 (57)	-35 (58)	0.705	-31 (73)	0.684	
Vitamin D, µg	Base	14.6 (10.5)	10.5 (6.1)		14.1 (12.5)		61-74y = 10, ≥ 75y = 20
	Δ 6-m	-4.7 (5.5)	-0.9 (5.3)	0.166	-2.2 (4.8)	0.572	
Vitamin E, α-TE †	Base	13.0 (5.7)	14.1 (11.0)		51.9 (3.6)		F = 8, M = 10
	Δ 6-m	-2.2 (4.2)	-2.2 (3.9)	0.119	-0.2 (2.9)	0.170	

*NNR* Nordic Nutrition Recommendations 2012 [35]; *MJ* Megajoule, *E%* Energy percent of total intake, *F* Females, *M* Males, *α-TE* α-tocopherol equivalent

^*^Statistically significant difference for 6-month follow-up with-in group compared to baseline, *p* < 0.05

^†^ Statistically significant difference between intervention groups and control group *p* < 0.05

### Nutrient intake

HDI mean score for each intervention and control group over time with 95% confidence intervals. *P* value indicates difference between the group compared to control group (estimated mean change from mixed linear regression model).

Food record data were obtained from Finland and Sweden. There were few differences between the groups at baseline; the intervention group had a lower fiber intake (β = -5.000, SE 2.401, *p* = 0.042), and the intervention + medical food group had a lower proportion of energy from alcohol (β = -0.027, SE 0.009, *p* = 0.049). The mean nutrient intake for all groups at baseline met the recommendations, except for saturated fatty acids (SFA) which was higher than the recommended (control 4E%, lifestyle intervention 3E%, lifestyle + medical food 2E% above the recommendation) [35]. In addition, the lifestyle intervention group had suboptimal fiber and potassium intake at baseline. Energy intake did not change between baseline and follow-up. However, there were some changes in the macronutrient composition. All groups showed a slight increase in fat intake and a slight decrease in protein intake. At follow-up, the lifestyle intervention group had a greater fiber intake (β = 6.136, SE 2.524, *p* = 0.018) and the lifestyle intervention + medical food group had a significantly greater intake of n-3 PUFA measured as proportion of the total energy intake than the control group (β = 1.777, SE = 0.615, *p* = 0.005).

Interestingly, all groups reported a decreased intake of vitamin C, vitamin D, and potassium at follow-up, even though neither of these within-group changes were significant. There were also no significant changes between the intervention groups and the control group.

There was no significant change in the nutrient density (unit/MJ) within either one of the intervention groups. However, in the control group a significant within group decrease was found for phosphate (β = -14.382, SE = 5.246, *p* = 0.013), niacin equivalents (β = -0.577, SE = 0.251, *p* = 0.040), vitamin D (β = -0.484, SE = 0.226, *p* = 0.039), and vitamin E (β = -0.252, SE = 0.097, *p* = 0.019).

## Discussion

Compared with that in the control group, dietary quality improved in both intervention groups over the study period when evaluated with indices reflecting overall diet pattern. Effect of the intervention was similar using both MEDAS screener to evaluate adherence to Mediterranean type of diet, and HDI to evaluate overall healthy diet There were few differences in macro- and micronutrient intake. However, the control group had significantly lower nutrient density for many nutrients, indicating a worsening diet. The results from this multimodal lifestyle intervention indicate that it is possible to improve dietary quality and prevent a decrease in nutrient intake with a dietary intervention among patients with prodromal AD. This can be important since they are at risk for malnutrition and dietary deficiencies, and preventing these changes could support maintenance of their health [43].

The FINGER trial showed that individualized dietary counseling for at-risk older adults can influence several dietary aspects and lead to long term improvement in dietary quality [29]. A large-scale, long-term dietary intervention among older individuals investigating the effects of the Mediterranean diet and olive oil or nuts showed a lasting improvement in dietary quality following the intervention [44]. Here we found fewer differences between the groups in terms of dietary intake; however, the group of participants was relatively small, and the trial had a shorter duration, which may have caused more variability and less statistical power to detect differences. There was an indication of decreased intake of several vitamins and minerals in relation to energy intake in the control group, which was not detected in the intervention groups.

The results from the 3-day food records are comparable to those of national population surveys conducted in their respective countries [45]. In general, the reported nutrient intake adhered to the NNR with the exception of a high SFA intake in all groups and a lower than recommended fiber intake in the intervention group [35] typically challenging also in healthy populations. Based on these findings, nutrient intake among patients with prodromal AD does not seem to differ from general population, which indicates there is still a possibility to support them maintain their nutritional status before the disease progresses. However, energy intake decreased across all groups without significant changes in macronutrient composition, possibly indicating some disease-related process. However, similar decline in energy intake has been shown in the FINGER study among healthier older adults [30], and it can also be related to age or participation in the trial.

Moreover, this decrease in energy intake was accompanied by a notable reduction in vitamin C intake. This decrease in vitamin C may be attributed to a lower consumption of fruits and vegetables. However, it is important to note that other nutrients typically found in fruits and vegetables, such as folate and fibers, remained relatively unchanged. Research findings have consistently shown that a significant proportion of older adults fail to adhere to the recommended daily intake of five servings of fruits and vegetables [46]. Moreover, a comprehensive meta-analysis has provided evidence linking a greater consumption of fruits and vegetables to a reduced prevalence of cognitive disorders [47]. Another reason could be seasonal variability, however, participants were recruited through the whole year and food records in close proximity to their study visits. Even though the participants in this study had decreased levels of vitamin C, they still reached the NNR of 80 mg/day [48].

Recent epidemiological evidence indicates that multiple dietary patterns, including the Mediterranean, DASH, MIND, and Nordic diets, have shown preventive effects against cognitive decline [19], mostly in cognitively healthy populations. The dietary indices investigated had a relatively small effect size, however, a moderate adherence to a healthy diet can have beneficial effects on brain health [49]. The use of a dietary index or score provides a comprehensive assessment of overall dietary quality and can be a valuable tool for evaluating the effectiveness of dietary interventions. However, it is important to acknowledge that relying solely on a score may overlook specific dietary components or individual variations.

One potential disadvantage of using a dietary index or score is the inherent complexity and subjectivity involved in assigning weights and cutoffs for different dietary components. Nonetheless, the use of such scores allows for a standardized evaluation of dietary quality and facilitates comparisons across studies. We observed similar improvements in both intervention groups regarding HDI and MEDAS, which is expected because they do share many items and are thus correlated. Despite both groups displaying similar slopes, the lifestyle intervention group showed a lower intercept, suggesting that the intervention had similar benefits regardless of the baseline diet.

There are limitations in this study. MIND-AD_mini_ is a pilot trial with the primary aim of determining the feasibility of and adherence to a multimodal lifestyle intervention for people with prodromal AD. Any other analysis should be seen as exploratory and as such, we did not correct for multiple comparisons, increasing the possibility of chance findings. On the other hand, the small sample size of a pilot trial may result in limited statistical power. Our food intake questionnaire was not validated in all countries participating in the trial, although it's suitability in international intervention settings had been tested [50, 51]. Furthermore, using memory recall tools for determining dietary intake, such as the FIQ, is prone to recall bias for a cognitively healthy population let alone for people with symptoms of cognitive decline [52]. This can be alleviated by using a shorter FIQ, obtaining help from a close study partner and checking by trained study personnel [53], which all were applied here. Nutrient intake data were not available from all countries, and the baseline food records already included medical food products, which makes it difficult to evaluate the effect of the product on total nutrient intake. Information on nutrient intake can still be important since diet is rarely studied in a population with prodromal AD. Based on our findings the group with medical food had greater intake of vitamin E and omega-3 fatty acids at baseline, but the product did not result in an overall greater intake of energy, as expected.

This study has several strengths. First, it specifically targets individuals with prodromal AD, which is a possibly crucial stage for intervention but where the potential for diet intervention is currently not well known. Second, it incorporates comprehensive dietary counseling as part of a multimodal lifestyle intervention, reflecting real-world conditions where multiple lifestyle factors are addressed simultaneously. Finally, this study assessed the feasibility of and adherence to dietary advice given over a relatively long six-month period, providing valuable insights into the practicality of implementing such interventions in real life.

Inadequate diet and poor nutrition have been identified as risk factors for various chronic diseases, including AD, but they can also modify the disease process after disease onset. Therefore, addressing dietary habits and improving dietary quality are essential for overall health and cognitive well-being in all stages from prevention to care. Our findings suggest that individuals in the prodromal stage of AD can modify their nutrient intake through dietary counseling, thereby potentially mitigating the risk of further cognitive decline.

## Conclusions

The MIND-AD_mini_ trial demonstrated that dietary counseling within a multimodal lifestyle intervention for individuals with prodromal AD is feasible and can lead to improvements in dietary quality. Future research should focus on long-term effects, individualized dietary counseling strategies, and the potential impact of improved dietary quality on cognitive outcomes in this population.

### Supplementary Information

Supplementary Material 1.

## Data Availability

The MIND-AD consortium is open to requests from external researchers for data collected in the trial. Requesters will be asked to submit a study protocol, including the research question, planned analysis, and data needed. The MIND-AD consortium will evaluate this plan (i.e., relevance of the research question, suitability of the data, quality of the proposed analysis, planned or ongoing MIND-ADmini analysis, and other matters) on a case-by-case basis and provide the data or reject the request. Shared data will encompass the data dictionary and deidentified participant data only. All analyses will be conducted in collaboration with and on behalf of the MIND-AD consortium. Access is subject to the MIND-AD legal framework. An access agreement will be prepared and signed by both parties.

## Abbreviations

AD: Alzheimer’s disease
BMI: Body Mass Index
CDR: Clinical Dementia Rating
DASH: Dietary Approaches to Stop Hypertension
DHA: Docosahexaenoic acid
DSM-IV: Diagnostic and Statistical Manual of Mental Disorders, Fourth Edition
E%: Energy percentage
FIQ: Food intake questionnaire
HDI: Healthy Diet Index
HDL: High-density lipoprotein
LDL: Low-density lipoprotein
MEDAS: Mediterranean Diet Adherence Screener
MIND: Mediterranean-DASH Intervention for Neurodegenerative Delay
MMSE: Mini Mental State Examination
MUFAs: Mono-unsaturated fatty acids
PUFAs: Poly-unsaturated fatty acids
RDI: Recommended daily intake
