# Supplementary material for: Nutrition guidance within a multimodal intervention improves diet quality in prodromal Alzheimer’s disease: Multimodal Preventive Trial for Alzheimer’s Disease (MIND-ADmini)
Source: Alzheimers Res Ther. 2024 Jul 3;16:147. doi: 10.1186/s13195-024-01522-8 (PMC11221015; doi:10.1186/s13195-024-01522-8)
Supplement: Supplementary file 1 — Supplementary Material 1. [file 13195_2024_1522_MOESM1_ESM.docx]

Nutrition guidance within a multimodal intervention improves diet quality in prodromal Alzheimer’s disease supplementary materials


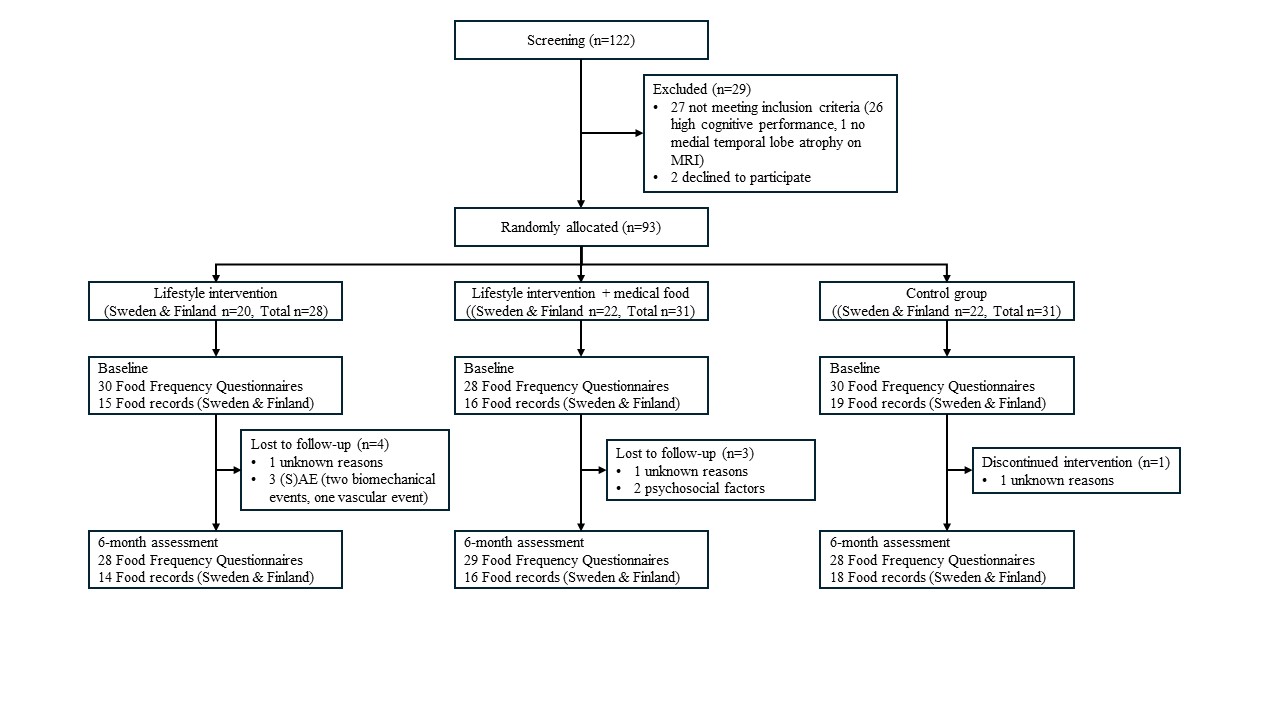
Supplementary figure 1
